# Intercarrier Interference Reduction in MC-CDMA System through Second Order Duobinary Coded Phase Rotated Conjugate Cancellation Scheme

**DOI:** 10.1371/journal.pone.0116326

**Published:** 2015-03-19

**Authors:** S. Chitra, N. Kumaratharan

**Affiliations:** 1 Department of Electronics and Communication Engineering, Rajalakshmi Engineering College, Chennai, Tamil Nadu, India; 2 Department of Information Technology, Sri Venkateswara College of Engineering, Chennai, Tamil Nadu, India; Beijing University, CHINA

## Abstract

Multi-carrier code division multiple access (MC-CDMA) technique is one of the strong candidates for next generation wireless mobile communication systems. Multi-carrier systems are very much sensitive to carrier frequency offset (CFO) results in intercarrier interference (ICI). To mitigate ICI without any spectral loss, a second order duobinary coded phase rotated conjugate cancellation algorithm is proposed in this paper. In the conventional phase rotated conjugate cancellation (PRCC) technique, one path carries the MC-CDMA signal with a phase spin of ϕ and the other path carries the conjugate of the first path signal with -ϕ phase spin. This artificial phase rotation allows the transmitter to tune the transmitted signals so that the ICI effects could be mutually cancelled at the receiver. Although the PRCC technique reduces the spectral efficiency, the limitation can be overcome by the joint second order duobinary coding scheme with PRCC technique. In the proposed method, the correlative coding between the binary symbols modulated on adjacent subcarriers is used to reduce the ICI without any spectral loss. Simulation results show that the proposed PRCC method provides better carrier to interference ratio (CIR) and bit error rate (BER) performances compared to the conventional conjugate cancellation (CC) technique.

## Introduction

Multi-carrier modulation (MCM) technique has been applied widely in wireless communication systems that support multimedia services such as audio, video, image and data. This is mainly due to its robustness to multi-path fading; high data rate transmission capability and easy implementation using Fast Fourier transform (FFT). The basic principle of MCM is dividing the high rate data stream into several low rate substreams. These substreams are transmitted through different subcarriers. Orthogonal frequency division multiplexing (OFDM) is one of the strong MCM techniques. The code division multiple access scheme (CDMA) allows many users to transmit their information on the same channel bandwidth using different spreading codes. MC-CDMA combines MCM with frequency domain spreading [1]. MC-CDMA system is susceptible to phase noise and carrier frequency offset (CFO) which leads to loss of orthogonality among the subcarriers causes an undesired effect called ICI that degrades the system performance [2, 3]. With several ICI cancellation schemes, self-cancellation is one of the simple and low complexity method compared to other techniques. The basic principle of ICI self-cancellation is the difference between ICI coefficients of two consecutive subcarriers is normally very small compared to the individual coefficients. Thus the ICI generated between the two sub-carriers gets mutually cancel each other. This method is suitable for multipath fading channels. The second method for ICI suppression in MC-CDMA system is the conjugate cancellation (CC) technique. The conventional CC technique employs two paths transmission algorithm in which, the first path carries the MC-CDMA signal and the second path carries the conjugate of the first path signal. The CIR performance of CC technique is inferior to normal MC-CDMA system in high frequency offset conditions. Third method which provides better CIR performance in both high and low frequency offset conditions is the phase rotated conjugate cancellation (PRCC) technique. In PRCC technique, one path carries the MC-CDMA signal with a phase rotation of ϕ and the other path carries the conjugate of the first path with a phase rotation of −ϕ. At the receiver, the frequency offset is estimated and feedback to the transmitter to calculate the phase rotation at the transmitter. PRCC technique uses the frequency shifting property of Fourier transform, that is the multiplication of signal with e^jϕ^ shift its frequency spectrum. The major drawback of all the two paths algorithm is the reduction in bandwidth efficiency as the same symbol occupies two subcarriers [4]. Duobinary coding is the correlative coding technique in which each coded symbol is calculated using correlation between successive symbols. The word ‘duo’ means to double the transmission capacity of the system. Duobinary coding technique changes the input sequence of uncorrelated binary digits into a sequence of correlated digits. The correlation between the adjacent binary digits is transmitted on each subcarrier. But the error propagation makes it unsuitable for efficient communication. In order to avoid the error propagation, a precoder (differential encoder) is used with duobinary coder. Unlike duobinary coding method, the overall transfer function of second order duobinary coding technique has no dc component. The spectral null at zero frequency is the desirable feature of second order duobinary coding scheme. This property is important since, many communication channels cannot transmit a dc component. To suppress the effect of ICI without reducing the spectral efficiency and to achieve better CIR performance in both low and high frequency offset conditions, a method that combines PRCC technique with second order duobinary coding is proposed.

This paper is organized as follows. Section 2 describes the general MC-CDMA system model and the second order duobinary coded phase rotated conjugate cancellation technique. MATLAB simulation results are discussed in section 3. The CIR and BER performance improvements are also discussed in this section.

## Experimental Methods

### MC-CDMA system model

The notion of MC-CDMA system is to spread the *i* ^th^ user data sequence $b_{k}^{i}$ (1 ≤ *i* ≤ *U* where *U* is the maximum number of users) using the spreading code $C_{k}^{i}$(0 ≤ *k* ≤ *N* − 1, where *N* is the number of subcarriers). The Walsh-Hadamard code, an orthogonal code [5] provides minimum multiple access interference (MAI) in the multipath fading channel. All the *U* user CDMA signals are subsequently mapped to N subcarriers using OFDM technique.

The joint CDMA signal of all the *U* users, for the *k* ^*th*^ chip of the spreading sequence is expressed as

$$S_{k}=\sum_{i=1}^{U}a_{k}^{i}C_{k}^{i}\;k=0,1,2,\ldots ,N-1$$ (1)

where *$a_{k}^{i}$* is the bipolar non-return to zero (BNRZ) representation of binary data sequence$b_{k}^{i}$. The data signal $a_{k}^{i}$ and spreading signal $C_{k}^{i}$ takes the value ±1.

The MC-CDMA signal which is the inverse FFT (IFFT) of CDMA signal is given by

$$s_{n}=1/N\sum_{k=0}^{N-1}S_{k}\exp(j2\pi nk/N)$$ (2)

where *n = 0*, *1*…, *N-1*. The cyclic prefix (CP) is interleaved and the signal is transmitted through the additive white Gaussian noise (AWGN) channel. The received signal in the presence of frequency offset [6] and AWGN (*w*_*1*_) is expressed as

$$r_{n}=s_{n}\exp(j2\pi n\varepsilon /N)+w_{1}$$ (3)

where *ε* is the normalized frequency offset, which is the carrier frequency offset normalized by spacing between the subcarriers.

At the receiver, the FFT of *p* ^*th*^ subcarrier is

$$\begin{array}{l} R_{p}=\sum_{n=0}^{N-1}\left\{\left[s_{n}\exp(j2\pi n\varepsilon /N)+w_{1}\right]\exp(-j2\pi np/N)\right\}\;\text{where}\;0\leq p\leq N-1\\ R_{p}=S_{p}I\left(-\varepsilon \right)+\sum_{k=0,k\neq p}^{N-1}S_{k}I\left(p-k-\varepsilon \right)+W_{1} \end{array}$$ (4)

where *W*_1_ is the FFT of *w*_1_ and $I\left(\varepsilon \right)=1/N\sum_{n=0}^{N-1}\exp(-j2\pi n\varepsilon /N)$

$$=\left(\sin\pi \varepsilon /N\sin\left(\frac{\pi \varepsilon }{N}\right)\right)\exp\left(j\pi \varepsilon \left(1-N/N\right)\right)$$

The First term in (4) is the desired term and the second term is the sum of interference due to the presence of frequency offset. Third term is the noise of AWGN nature [7]. The term *I*(*p* − *k*) in (4) is zero, when the carriers are orthogonal.

The output of desired user ‘*i*’ on the *p* ^th^ subcarrier after despreading is given by

$$Y_{p}^{i}=a_{p}^{i}I\left(-\varepsilon \right)+\sum_{k=0,k\neq p}^{N-1}S_{k}I(p-k-\varepsilon )+W_{1}$$ (5)

### Second order duobinary coded phase rotated conjugate cancellation technique

**Fig 1:**
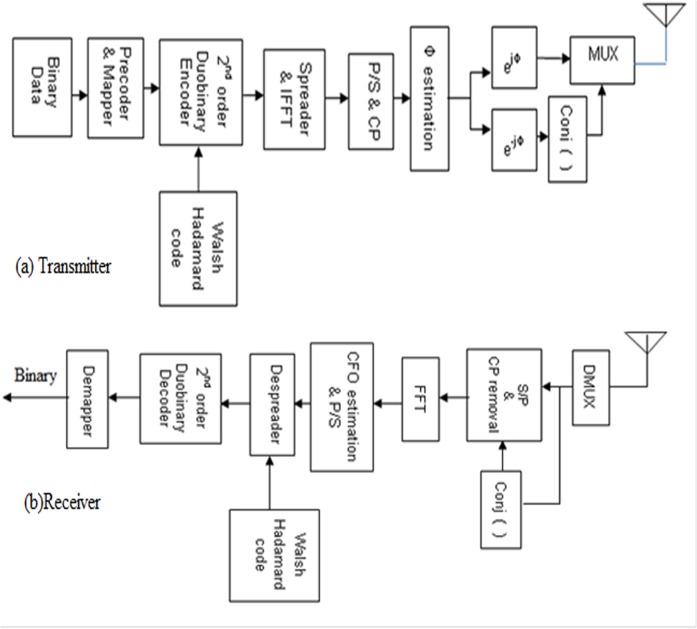
Block diagram of Second order Duobinary coded PRCC Technique. (a) Transmitter (b) Receiver.

The basic principle of duobinary coder is, to add an intersymbol interference (ISI) to the transmitted signal in a controlled manner and compensating its effect at the receiver. The signaling rate of duobinary coding scheme is twice the transmission bandwidth of the channel. It doubles the transmission capacity of the system with better error rate performance. The duobinary coding technique [8, 9] involves a correlation span of two binary digits (*a*_*k*_,*a*_*k*−1_). The PRCC technique with second order duobinary coding is implemented and analyzed in this paper. The second order duobinary coding technique employs the correlation between *a*_*k*_ and *a*_*k*−2_. This is achieved by subtracting the input binary digits spaced 2T_b_ seconds apart, where T_b_ is the bit duration. The simplified block diagram of second order duobinary coded MC-CDMA system with PRCC is shown in Fig. 1.

To eliminate the possibility of error propagation [10], a precoder is used prior to the second order duobinary coder. The precoder output is given by

$$d_{pk}=b_{k}⊕d_{pk-2}$$ (6)

The sequence *d*_*pk*_ is applied to the second order duobinary conversion filter. The filtered output is given by

$$m_{k}=a_{pk}-a_{pk-2}$$ (7)

Where $a_{pk}=\left\{ \begin{array}{ll} 1 & \text{if}\;d_{pk}=1\\ -1 & \text{if}\;d_{pk}=0 \end{array}\right.$

Then the second order duobinary coder output is spread by the Walsh Hadamard spreading sequence *C*_*k*_. The signal after spreading is given by

$$X_{k}=m_{k}\cdot C_{k}$$ (8)

The MC-CDMA signal at the IFFT output [11] is expressed as

$$\begin{array}{l} x_{n}=1/N\sum_{k=0}^{N-1}X_{k}\;\exp(j2\pi kn/N)\\ x_{n}=1/N\sum_{k=0}^{N-1}m_{k}C_{k}\exp(j2\pi kn/N) \end{array}$$ (9)

In second order duobinary coded PRCC technique, the first path signal is phase rotated by ϕ and the second path signal is the conjugate of first path signal with a phase rotation of −ϕ. The transmitted signals at two transmission paths are

$$y_{n}^{\left(1\right)}=x_{n}\exp(j\phi )\;\text{and}\;y_{n}^{\left(2\right)}={\left(x_{n}\exp(-j\phi )\right)}^{*}$$ (10)

At the receiver, the first path received signal in the presence of frequency offset ε is given by

$$z_{n}^{\left(1\right)}=y_{n}^{\left(1\right)}\exp(j2\pi n\varepsilon /N)+w_{1}\;\text{where}\;\;n=0,1,\ldots ,N-1$$

where *n = 0*, *1*,…, *N-1*.

The signal at the second path is given by

$$z_{n}^{\left(2\right)}=y_{n}^{\left(2\right)}\exp(j2\pi n\varepsilon /N)+w_{2}$$ (11)

Where *w*_1_ and *w*_2_ are additive white Gaussian noises in the first and second path respectively. At the receiver the two path signals are separated and converted into frequency domain using FFT. The FFT output of first path and the conjugate of the second path for *p* ^th^ subcarrier are expressed as

$$\begin{array}{l} Z_{p}^{\left(1\right)}=\sum_{n=0}^{N-1}z_{n}^{\left(1\right)}\exp(-j2\pi np/N)\\ \;\;\;\;\;\;\;\;=1/N\sum_{k=0}^{N-1}X_{k}\sum_{n=0}^{N-1}\exp(j\phi )\exp(-j2\pi n(p-k-\varepsilon )/N)+W_{1}\\ Z_{p}^{\left(1\right)}=\sum_{k=0}^{N-1}X_{k}(\exp(j\phi )I(p-k-\varepsilon ))+W_{1} \end{array}$$ (12)

Similarly

$$Z_{p}^{\left(2\right)}=\sum_{k=0}^{N-1}X_{k}\left(\exp\left(-j\phi \right)I\left(p-k+\varepsilon \right)\right)+W_{2}$$ (13)

Where *W*_1_ and *W*_2_ are FFT of *w*_1_ and *w*_2_.The receiver output is the average of two FFT outputs, and is expressed as

$$Z_{av}=\frac{1}{2}(Z_{p}^{\left(1\right)}+Z_{p}^{\left(2\right)})$$

The FFT signal after despreading is given by

$$Z_{de}=Z_{av}C_{k}$$

The decision rule used by second order duobinary decoder is

$$b_{k}=\left\{ \begin{array}{ll} 0 & if\left|Z_{de}\right|<1\\ 1 & if\left|Z_{de}\right|>1 \end{array}\right.$$ (14)

### CIR performance improvement

The CIR of conventional CC technique [12] is expressed as

$${\text{CIR}}_{\text{CC}}=\frac{\left|I(-\varepsilon )+I(+\varepsilon )\right|}{\sum_{k=1}^{N-1}\left|I(k-\varepsilon )+I(k+\varepsilon )\right|}$$ (15)

The received signal *Z*_*T*_ can be expressed as sum of desired signal and interference signal.

$$Z_{T}=X_{p}\left\{\exp(j\phi )I(-\varepsilon )+\exp(-j\phi )I(\varepsilon )\right\}+\sum_{k=0,k\neq p}^{N-1}X_{k}\{\exp\left(j\phi \right)I\left(p-k-\varepsilon \right)+\exp\left(-j\phi \right)I\left(p-k+\varepsilon \right)\}+W_{1}+W_{2}$$ (16)

The first term in Eq. (16), represents the desired information and the second term represents the sum of interference from *k* ^th^ subcarrier to *p* ^th^ subcarrier. Third term corresponds to AWGN signal. The CIR of conventional PRCC scheme [13] is given by

$${\text{CIR}}_{\text{PRCC}}=\frac{{\left|\exp(j\phi )I(-\varepsilon )+\exp(-j\phi )I(+\varepsilon )\right|}^{2}}{\sum_{k=1}^{N-1}{\left|\exp(j\phi )I(k-\varepsilon )+\exp(-j\phi )I(k+\varepsilon )\right|}^{2}}$$ (17)

The PRCC technique with second order duobinary coding scheme improves the CIR performance. The optimum phase rotation for a given frequency offset ε is calculated by finding the phase corresponding to maximum CIR value. From (17), the numerator is expressed as

$$\begin{array}{l} =(\exp(j\phi )I(-\varepsilon )+\exp(-j\phi )I(\varepsilon ))\times (\exp(-j\phi )I^{*}(-\varepsilon )+\exp(j\phi )I^{*}(\varepsilon ))\\ ={\left|I(-\varepsilon )\right|}^{2}+\exp(j2\phi )I(-\varepsilon )I^{*}(\varepsilon )+\exp(-j2\phi )I(\varepsilon )I^{*}(-\varepsilon )+{\left|I(\varepsilon )\right|}^{2} \end{array}$$

where I(−ε)=I(ε)exp(−j2πε(1−N)/N) and${\left|I(-\varepsilon )\right|}^{2}={\left|I(\varepsilon )\right|}^{2}$.

Thus the term |exp(*j*ϕ)*I*(− ε) + exp(− *j*ϕ)*I*(+ ε)|^2^ is expressed as

$$2{\left|I(\varepsilon )\right|}^{2}+2{\left|I(\varepsilon )\right|}^{2}\cos(2\phi +2\pi \varepsilon (N-1)/N)$$ (18)

The denominator term in Eq. (17), can be expressed as

$$\sum_{k=1}^{N-1}{\left|I(k-\varepsilon )\right|}^{2}+{\left|I(k+\varepsilon )\right|}^{2}+2\left|I(k-\varepsilon )I(k+\varepsilon )\right|\cos(2\phi +2\pi \varepsilon (N-1)/N)$$ (19)

The optimum phase rotation is obtained by

$$\partial CIR_{PRCC}(\varepsilon ,\phi )/\partial \phi =0$$ (20)

Using (23) and (24) in (22) and the resultant CIR expression is differentiated with respect to ϕ and equating to zero.

$$\begin{array}{l} 4\sin(2\phi +2\pi \varepsilon (N-1)/N)\times \left\{\begin{array}{l} \left|I(\varepsilon )\right|\sum_{k=1}^{N-1}{\left|I(k-\varepsilon )\right|}^{2}+{\left|I(k+\varepsilon )\right|}^{2}+2\left|I(k-\varepsilon )I(k+\varepsilon )\right|\cos(2\phi +2\pi \varepsilon (N-1)/N)-\\ (2{\left|I(\varepsilon )\right|}^{2}+2{\left|I(\varepsilon )\right|}^{2}\cos(2\phi +2\pi \varepsilon (N-1)/N))\times \left|I(k-\varepsilon )I(k+\varepsilon )\right| \end{array}\right\}=0\\ \sin(2\phi +2\pi \varepsilon (N-1)/N)=0 \end{array}$$

Then

2ϕ+2πε(N−1)/N=yπor0

where y = 0, 1, 2…

2ϕ+2πε(N−1)/N=0

From the above equation the optimum phase rotation for maximum CIR is given by

$$\phi_{opt}=-\pi \varepsilon (N-1/N)$$ (21)

To obtain the CIR of the proposed method, the average carrier power *E*[|*r*_*k*_|^2^] and the average interference power *E*[|*I*_*k*_|^2^] should be calculated separately. The desired signal for second order duobinary coded PRCC is given as

$$r_{k}=\exp(j\phi )m_{k}C_{k}I(-\varepsilon )+\exp(-j\phi )m_{k}C_{k}I(\varepsilon )$$ (22)

The interference signal is given as

$$I_{k}=\sum_{k=1,k\neq p}^{N-1}\exp(j\phi )m_{k}C_{k}I(p-k-\varepsilon )+\sum_{k=1,k\neq p}^{N-1}\exp(-j\phi )m_{k}C_{k}I(p-k+\varepsilon )$$ (23)

where *I*(*p* – *k* ± ε) = sin(π(*p* – *k* ± ε)/*N* sin(π (*p* – *k* ± ε)/*N*) exp(*j* π (*p* – *k* ± ε)(1 – *N*)/*N*

The average carrier power is

$$E\left[{\left|r_{k}\right|}^{2}\right]=E\left[{\left|\exp(j\phi )m_{k}C_{k}I(-\varepsilon )+\exp(-j\phi )m_{k}C_{k}I(\varepsilon )\right|}^{2}\right]$$ (24)

Where the value of $C_{k}^{2}$ is always 1 and the sequence *a*_*k*_ satisfies the independence condition. Therefore

$$E\left[a_{k}a_{k-p}\right]=\left\{ \begin{array}{l} E[{\left(a_{k}\right)}^{2}]\text{ for k = p }\\ 0\text{ for k}\neq \text{p} \end{array}\right.$$

For binary phase shift keying (BPSK) signal, the assumption is *E*[*a*_*k*_] = *E*[*a*_*k*−2_] = 0 Therefore

$$\begin{array}{l} E\left[\left(m_{k}^{2}\right)\right]=E\left[{\left(a_{k}-a_{k-2}\right)}^{2}\right]=2E\left[{\left(a_{k}\right)}^{2}\right]\\ E\left[{\left|r_{k}\right|}^{2}\right]=2E\left[{\left(a_{k}\right)}^{2}\right]\left[{\left|\exp(j\phi )I(-\varepsilon )+\exp(-j\phi )I(+\varepsilon )\right|}^{2}\right] \end{array}$$ (25)

The average ICI power E[|*I*_*k*_|^2^] is given as

$$\begin{array}{c} E\left[{\left|I_{k}\right|}^{2}\right]=E\left[{\left|\sum_{k=1,k\neq p}^{N-1}\exp(j\phi )m_{k}C_{k}I(p-k-\varepsilon )+\sum_{k=1,k\neq p}^{N-1}\exp(-j\phi )m_{k}C_{k}I(p-k+\varepsilon )\right|}^{2}\right]\\ =\overset{N-1}{\underset{\begin{array}{l} k=1\\ k\neq p \end{array}}{\sum }}\overset{N-1}{\underset{\begin{array}{l} d=1\\ d\neq k \end{array}}{\sum }}\left\{\begin{array}{l} (\exp(j\phi )I(p-k-\varepsilon )+\exp(-j\phi )I(p-k+\varepsilon ))\times \\ \left(\exp(-j\phi )I^{*}(p-d-\varepsilon )+\exp(j\phi )I^{*}(p-d+\varepsilon ))E[m_{k}m_{d}\right] \end{array}\right\} \end{array}$$

To calculate the average ICI power the correlation between m_k_ and m_k±2_ is considered.

$$E\left[m_{k}m_{d}\right]=\left\{ \begin{array}{ll} \text{E}\left[{\text{(a}}_{\text{k}}{\text{-a}}_{\text{k-2}}{\text{)(a}}_{\text{d}}{\text{-a}}_{\text{d-2}}\text{)}\right]\text{=2E}\left[{{\text{(a}}_{\text{k}}\text{)}}^{\text{2}}\right] & \text{for}\;k=d\\ -E\left[{\left(a_{k}\right)}^{2}\right] & \text{for}\;d=k\pm 2\\ 0\;\;\;\;\;\;\;\;\;\;\;\;\;\;\;\;\;\;\;\;\;\;\;\;\;\;\;\;\;\;\;\;\;\;\;\;\text{otherwise} & \end{array}\right.$$

The CIR of PRCC scheme with second order duobinary coding scheme is given by

$$\text{CIR }=\frac{{\left|\exp(j\phi )I(-\varepsilon )+\exp(-j\phi )I(+\varepsilon )\right|}^{2}}{\sum_{k=1}^{N-1}{\left|\exp(j\phi )I(k-\varepsilon )+\exp(-j\phi )I(k+\varepsilon )\right|}^{2}-L_{I}}$$ (27)

where

$$L_{I}=1/2\sum_{k=3}^{N-1}\left[\begin{array}{l} {}[\exp(j\phi )I(k-\varepsilon )+\exp(-j\phi )I(k+\varepsilon )][\exp(-j\phi )I^{*}(k-\varepsilon -2)+\\ \exp(j\phi )I^{*}(k+\varepsilon -2)]+[\exp(j\phi )I(k-\varepsilon -2)+\exp(-j\phi )I(k+\varepsilon -2)]\\ \left[\exp(-j\phi )I^{*}(k-\varepsilon )+\exp(j\phi )I^{*}(k+\varepsilon )\right] \end{array}\right]$$

## Results and Discussion

The MC-CDMA system is analyzed with BPSK modulation and Walsh Hadamard spreading code. The CIR and BER performance of second order duobinary coded PRCC scheme is compared with the normal MC-CDMA system and MC-CDMA system with CC technique. The Simulation parameters are listed in Table 1.

**Table 1:** Simulation Parameters.

Parameter	Value
Number of symbols	5200
Number of subcarriers	4,8,16,32
Number of users	10
Spreading code	Walsh Hadamard
Frequency offset	0.1,0.2,0.3,0.4,0.5
Channel	AWGN, Rayleigh

The performance evaluation for simulation results is listed in Table 2.

**Table 2:** Performance Evaluation of the Proposed Technique.

Performance	MC-CDMA	MC-CDMA with CC	Proposed PRCC
E_b_/N_o_ for BER of 10^−3^	10.8 dB	10.2 dB	8.4dB
Maximum CIR	40 dB	76 dB	92dB

**Fig 2:**
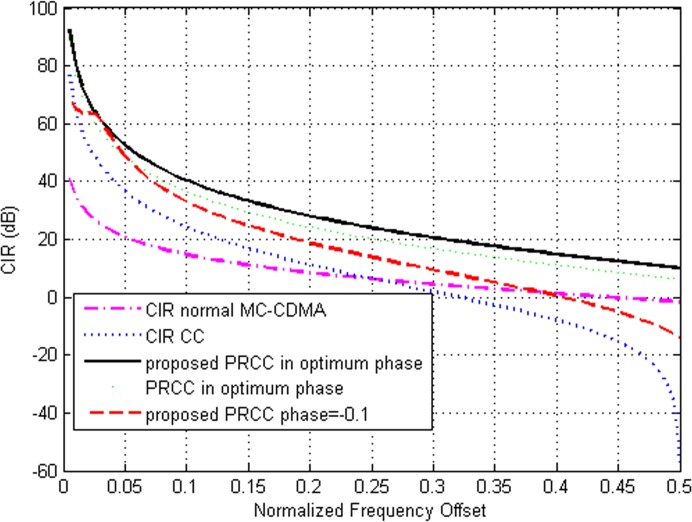
CIR Comparison of Second order Duobinary coded PRCC with Normal MC-CDMA, CC and Conventional PRCC.

Fig. 2 illustrates the CIR performance comparison of second order duobinary coded PRCC technique with other ICI cancellation schemes in different normalized frequency offset values. The graph clearly shows that the maximum CIR obtained in proposed technique is 92 dB and the conventional CC technique with second order duobinary coding is 76 dB. The CIR obtained in the PRCC technique with optimum phase is 88.5 dB and in the normal MC-CDMA system is 40 dB. The CIR value obtained in second order duobinary coded PRCC is high compared to other ICI cancellation techniques.

**Fig 3:**
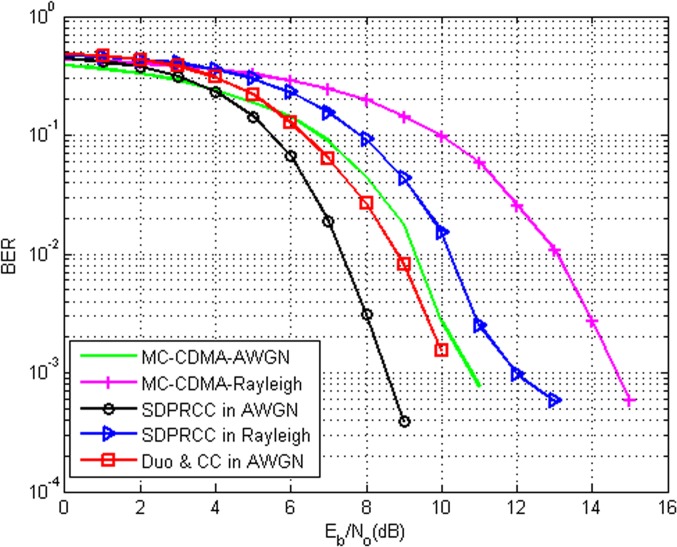
BER Comparison of Second order Duobinary coded PRCC in AWGN and Rayleigh channel with MC-CDMA and CC Technique with ε = 0.44.

Fig. 3 compares the BER performance of the proposed PRCC technique with other ICI cancellation schemes under various E_b_/N_o_ values. The analysis is carried out with the normalized frequency offset of 0.44 and with the data symbols of 5200. The graphs show that for the same number of data symbols, the BER of 10^−3^ is achieved at energy per bit per noise spectral density (E_b_/N_o_) of around 8.4 dB in the proposed technique and to that of 10.2 dB in conventional CC method. Further the graph illustrates the performance of the proposed technique in AWGN channel and Rayleigh fading channel. The result shows that at ε = 0.44, to achieve the BER of around 10^−3^, the proposed method needs 8.4 dB in AWGN channel and to achieve the same performance in Rayleigh fading channel the system needs 12 dB.

**Fig 4:**
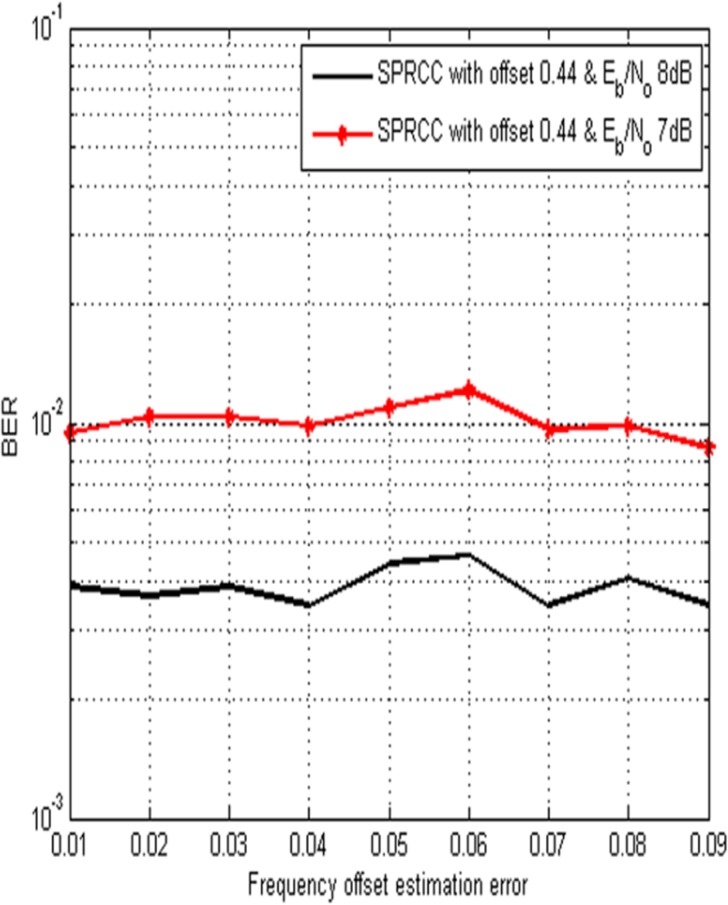
BER performance of the second order duobinary coded PRCC in the presence of frequency offset estimation error.

Fig. 4 illustrates the performance comparison interms of BER Vs. estimation error. It shows that the proposed method provides better BER performance even in the presence of offset estimation error. The BER performance of the proposed system is not much sensitive to frequency offset estimation errors due to the optimal phase rotation at the transmitter. In the proposed method, the frequency offset information at the receiver is estimated and fed back to the transmitter to introduce the phase rotation ϕ at the transmitter.

At the receiver the frequency offset is estimated using maximum likelihood estimation (MLE) algorithm, where in MLE, the frequency offset is estimated by repeating the same data frame twice and comparing the received signals at the receiver [14,15]. Two received signals are not being the same, when there is an offset. However MLE gives the estimate of relative frequency offset irrespective of E_b_/N_o_ values. The maximum estimation error considered for simulation is 0.09 with E_b_/N_o_ values of 7 dB and 8 dB.

**Fig 5:**
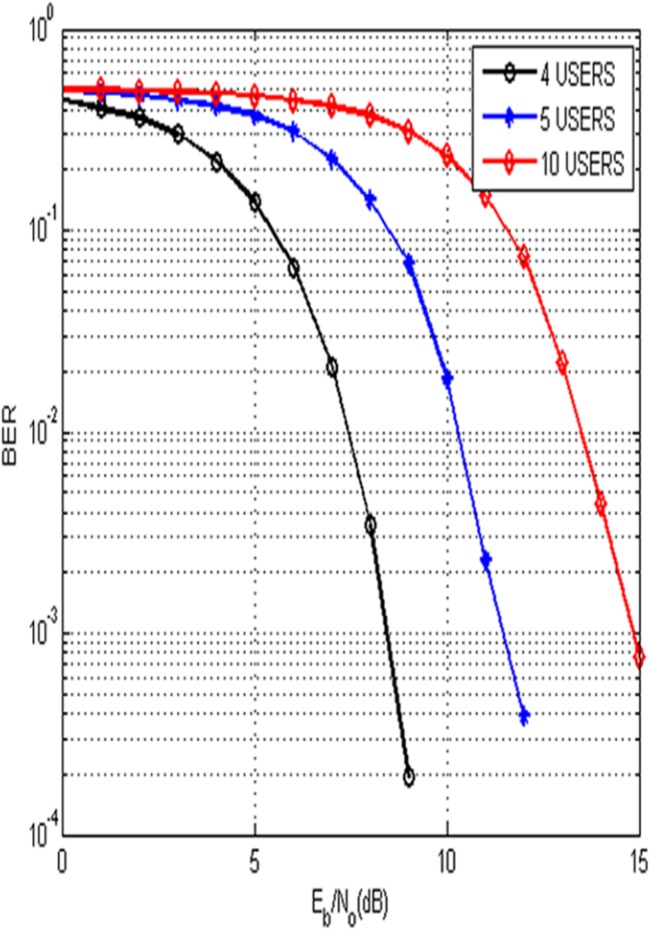
BER Performance of the Proposed Technique for Multiple Users.

Fig. 5 illustrates the BER comparison of the proposed technique with multiple users such as 4, 5 and 10. Results show that the simultaneous users experience little difference in their BER performance. This is due to the fact that when the number of simultaneous users is increased, the BER performance is gradually degraded. The graph shows that the BER of 10^−3^ is achieved at E_b_/N_o_ of 9 dB for 4 users, 12 dB for 5 users and at 15 dB for 10 simultaneous users accessing the channel.

**Fig 6:**
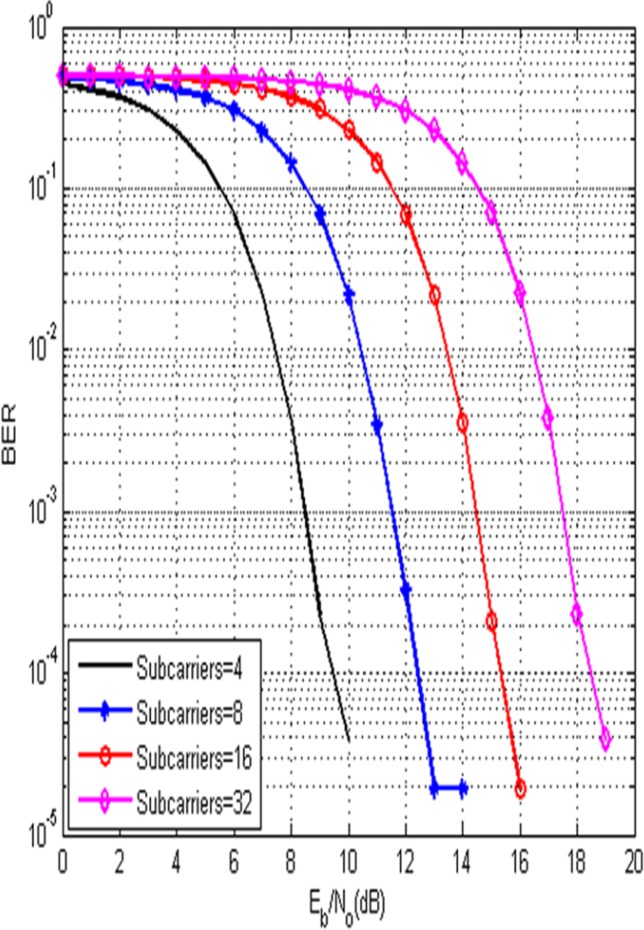
BER Performance of the Proposed Technique for different subcarriers.

Fig. 6 compares the performance of the proposed technique under various numbers of subcarriers with the data symbols of 52000. To achieve the BER of 10^−4^, the required E_b_/N_o_ are 9, 12, 15 and 18 dB for the subcarriers of 4,8,16 and 32. The curves clearly show that the BER is increased when the number of subcarriers is increased. It is mainly due to the fact that the amount of ICI power depends on the number of subcarriers. For large number of subcarriers, the ICI produced by the system is more which in turn increases the BER.

## Conclusion

The second order duobinary coded PRCC technique is proposed in this paper. The simulation results show that the CIR and BER performance of the proposed technique is better than the conventional CC technique in both low and high frequency offset conditions. Due to the artificial phase rotation and duobinary coding algorithm at the transmitter, the proposed technique reduces the ICI without reducing the spectral efficiency. Whenever the interference is reduced, obviously more number of users can be accommodated in the allocated spectrum which in turn increases the capacity of MC-CDMA system. Thus the proposed technique shall meet the requirements of future mobile radio communication systems by allowing many users to get the services simultaneously without any performance degradation. Further the work may be extended by using higher level modulation schemes and introducing the frequency offset compensation at the receiver side.
